# A Novel Factor Xa-Inhibiting Peptide from Centipedes Venom

**DOI:** 10.1007/s10989-013-9353-0

**Published:** 2013-06-19

**Authors:** Yi Kong, Yu Shao, Hao Chen, Xin Ming, Jin-Bin Wang, Zhi-Yu Li, Ji-Fu Wei

**Affiliations:** 1 School of Life Science & Technology, China Pharmaceutical University, 24 Tong Jia Xiang, Nanjing, 210009 People’s Republic of China; 2 State Key Laboratory of Natural Medicines, China Pharmaceutical University, Nanjing, 210009 People’s Republic of China; 3 Research Division of Clinical Pharmacology, The First Affiliated Hospital with Nanjing Medical University, 300 Guangzhou Road, Nanjing, 210029 China; 4 School of Pharmacy, China Pharmaceutical University, 24 Tong Jia Xiang, Nanjing, 210009 People’s Republic of China; 5 Division of Molecular Pharmaceutics, UNC Eshelman School of Pharmacy, The University of North Carolina at Chapel Hill, Chapel Hill, NC 27599 USA

**Keywords:** *Scolopendra subspinipes mutilans* venom, FXa inhibitor, Peptide, Anticoagulation

## Abstract

Centipedes have been used as traditional medicine for thousands of years in China. Centipede venoms consist of many biochemical peptides and proteins. Factor Xa (FXa) is a serine endopeptidase that plays the key role in blood coagulation, and has been used as a new target for anti-thrombotic drug development. A novel FXa inhibitor, a natural peptide with the sequence of Thr-Asn-Gly-Tyr-Thr (TNGYT), was isolated from the venom of *Scolopendra subspinipes* *mutilans* using a combination of size-exclusion and reverse-phase chromatography. The molecular weight of the TNGYT peptide was 554.3 Da measured by electrospray ionization mass spectrometry. The amino acid sequence of TNGYT was determined by Edman degradation. TNGYT inhibited the activity of FXa in a dose-dependent manner with an IC_50_ value of 41.14 mg/ml. It prolonged the partial thromboplastin time and prothrombin time in both in vitro and ex vivo assays. It also significantly prolonged whole blood clotting time and bleeding time in mice. This is the first report that an FXa inhibiting peptide was isolated from centipedes venom.

## Introduction

Centipedes (*Scolopendra subspinipes mutilans* L. Koch) are predatory, elongated and dorsoventrally flattened arthropods, which belong to the Chilopod class (Negrea and Minelli 1995). In China, centipedes have been used for thousands of years as a traditional Chinese medicine for treating the diseases, such as stroke-induced hemiplegia, apoplexy, epilepsy, tetanus, whooping cough, tuberculosis, scald burns, and pyocutaneous disease. Especially, they have been prescribed for treating cardiovascular diseases in Korea, China and other countries in East Asia for several hundred years (Pemberton 1999).

Centipede venoms were released from the venom glands connecting to the first pair forceps to kill prey and defend against its predators (Rates et al. 2007). The venoms are a natural pool of proteins, peptides and enzymes with a rich variety of biologic activities. Recent studies have showed that centipede venoms contain more than 500 proteins and peptides with the following pharmacological properties: platelet aggregating activity, anticoagulant activity, phospholipase A2 activity, trypsin inhibiting activity, voltage-gated potassium channel activities, voltage-gated sodium channel activities, voltage-gated calcium channel activities (Liu et al. 2012; Yang et al. 2012).

Coagulation factors are the main targets for anti-thrombotic drug development (Simkhada et al. 2012). Among these coagulation factors, Factor Xa (FXa), which is a serine endopeptidase, plays the key role in the blood coagulation, because it functions at the convergence point of the extrinsic and intrinsic pathways for blood coagulation (Gan et al. 2009). There have been reports of discovery of FXa inhibitors by isolating proteins or peptides from natural sources, since antistasin, a 119-amino acid protein, was isolated from the salivary glands of the Mexican leech *Haementeria officinalis* in 1987 (Dunwiddie et al. 1989). A 60-amino acid anticoagulant peptide was purified from extracts of the soft tick *Ornithodoros moubata* and was shown to inhibit FXa only (Waxman et al. 1990). In another report, a FXa-inhibiting peptide called Draculin was isolated from the saliva of Vampire bats (*Desmodus rotundus*) (Fernandeza et al. 1999). In addition, *Ac*-AP-12 (Jiang et al. 2011), Nymphal FXa inhibitor (Batista et al. 2010), and AduNAP4 (Gan et al. 2009) are the peptides with FXa inhibiting activity. However, FXa inhibitor has not been isolated from centipede venom.

In this study, we isolated a novel peptide, with the sequence of TNGYT, from *S. subspinipes mutilans* venom, which can inhibit FXa activity. Moreover, this peptide was shown to resist coagulation with in vitro assay and in mice. To our best knowledge, this is the first report demonstrating that a peptide purified from centipede venom can inhibit FXa activity.

## Materials and Methods

### Materials

Adult *S. subspinipes mutilans* L. Koch (both sexes, *n* = 1,000) was purchased from Qiancheng centipede breeding field (Jinhua, Zhejiang Province, China).

Sephadex G-50 was obtained from Pharmacia (Uppsala, Sweden). Purified human FXa and chromogenic substrate of FXa (S2222) were purchased from HYPHEN BioMed (Neuvile sur Oise, France). Mice of both sexes were from Qinglongshan animal center (Nanjing, Jiangsu Province, China). All the animal protocols were approved by the Animal Care and Use Committee at China Pharmaceutical University. All other chemicals and reagents were at analytical grade.

### Venom Extraction

Venom was collected manually by stimulating the venom glands in the first pair forceps of centipedes using the multi-purpose electrical instrument with the frequency at 7.8 ms (i.e. 128 Hz), the voltage at 10–20 V, and the pulse width at 2–4 ms. Each milking occurred 1 week after the previous milking. Venoms were stored at −20 °C until further use.

### Anticoagulant Peptide Purification

The collected venom (0.5 g) was diluted in 20 ml phosphate buffered saline (PBS, pH 6.0), and then centrifuged at 10,000 rpm for 8 min. The supernatant was applied to a SephadexG-50 column (26 mm × 100 cm), equilibrated with PBS (pH 6.0) and eluted with the same buffer at a flow rate of 0.6 ml/min. The fractions were collected at 10 min intervals. The absorbance of the elutes was monitored at 214 nm. The fractions were pooled according to the absorbance. The activated partial thromboplastin time (aPTT) and prothrombin time (PT) were evaluated for each fraction. The fraction 2 which showed prolonged aPTT and PT activities was further separated by reverse-phase high performance liquid chromatography (RP-HPLC) (Bio-Rad Duoflow System, USA) on a Lichrospher C18 column (10 mm × 250 mm; Hanbon, China) with a linear gradient elution conditions using acetonitrile as the organic modifier and trifluoroacetic acid (TFA) as the volatile buffer. Eluent A consisted of 0.1 % TFA in 10 % acetonitrile (v/v), eluent B of 0.1 % TFA in 90 % acetonitrile (v/v). Gradient elution was carried out according to the following process: 0–10 min, B 0 %; 10–50 min, B 0–100 %. The flow rate was at 1 ml/min. The UV absorbance was monitored at 214 nm. The fractions that prolonged aPTT and PT (2–6) were collected and further purified by another C18 column (4.6 mm × 250 mm; Hanbon, China) eluted as the same condition above. The main peak was pooled and stored at −20 °C.

### Determination of Molecular Mass and Peptide Sequence

The molecular mass of the purified peptide was determined by electrospray ionization mass spectrometry (ESI-MS) (Agilent6500, Agilent, USA) with the following conditions: ESI^+^ ion source, spray voltage was 3,500 V, fragmentator voltage was 120 V, capillary temperature was 365 °C, the pressure of ESI nebulizing gas (N_2_) was 275.79 kPa (40 psi), and the flow rate of drying gas (N_2_) was 10.00 l/min. Complete peptide sequencing was performed using Edman degradation method on an Applied Biosystems pulsed liquid-phase sequencer, model 491.

### Peptides Synthesis

The peptide used for the following bioactivity assays was synthesized by the Fmoc (*N*-[9-fluorenyl]-methoxycarbonyl) chemistry in solid-phase synthesis. Usually, peptides are synthesized from the carbonyl group side (C-terminus) to amino group side (N-terminus) of the amino acid chain. The solid supports were preloaded with 2-chlorotrityl chloride resin for C-terminal acid in this synthesis. Protected amino acids were coupled by in situ activation with diisopropylethylamine (DIEA) and *N*-hydroxybenzotriazole (HOBt). Then dimethylformamide (DMF) with 20 % piperidine was performed in deprotection for 20 min. Cleavage of the peptide from the resin was performed by reagent (95 % TFA/2.5 % triisopropylsilane (TIS)/2.5 % water) for 1 h. The peptide was purified by preparative reverse-phase HPLC on a C18 column (20 × 250 mm). Elution was performed using a linear gradient (0–50 % B in 20 min) of 0.1 % TFA in 10 % acetonitrile (A) and 0.1 % TFA in 90 % acetonitrile (B) at a flow rate of 2 ml/min. The absorbance was monitored at 214 nm. The main peak was pooled, dried in vacuum, lyophilized, and stored at −20 °C. The purity was analyzed by HPLC and ESI–MS.

### PT and aPTT Clotting Assays

Blood was drawn by Eyeball from ICR mice. The blood was separately centrifuged at 2,500×*g* for 10 min to obtain platelet poor plasma (PPP). For the in vitro aPTT assay, normal citrated PPP (50 μl) was incubated with sample solution (8, 6, 4, 2, 1 mg/ml, previously diluted in PBS, pH 8.34, 50 μl) and 100 μl aPTT reagent for 3 min at 37 °C. Clotting time was immediately recorded after the addition of 100 μl of 20 mM CaCl_2_. For the in vitro PT assay, normal citrated PPP (25 μl) was incubated with sample solution (8, 6, 4, 2, 1 mg/ml, previously diluted in PBS, pH 8.34, 25 μl) for 3 min at 37 °C. Clotting time was immediately recorded after the addition of 50 μl of PT reagent (Majdoub et al. 2009). All coagulation assays were performed in triplicates. Heparin was used as a positive control, and PBS (pH 8.34) as model control. For ex vivo coagulation assays, samples were dissolved in saline and were intravenously injected (10 or 20 mg/kg) in ICR mice (6 animals/group). Five and 24 h after drug administration, blood was collected (drawn by eyeball), immediately centrifuged (3,000 rpm, 15 min), and the plasma was used to measure the aPTT and PT as previously described (Batista et al. 2010).

### Clotting Time In Vivo

Whole blood clotting time (CT) in mice was measured by capillary glass tube method (Li et al. 2004). Mice (18–22 g body weight) were divided into four groups (both sexes, six per group). Two groups were received intravenous injection of 20 and 10 mg/kg body weight of the purified peptide for four consecutive days. Other groups were received normal saline and 20 mg/kg body weight of heparin (the positive control), respectively. Fifteen minutes after the last administration (positive control after 2 h), blood samples were collected through the retro-orbital plexus with a glass capillary and kept on a slide to allow for clotting. Stirring the blood with a dry needle every 30 s until needle wire can provoke a fibrous protein, when is defined as clotting time.

### Bleeding Time In Vivo

The mice (both sexes, 18–22 g) were randomly divided into four groups. The samples were intravenously injected (10 or 20 mg/kg) in ICR mice (6 animals/group) once a day in four consecutive days. Fifteen minutes after the last administration (positive control after 2 h), the mice tail was marked with a tag in the distance of 3 mm to after peak, then cut in the mark. Then the tip of the tail was immersed in saline at 37 °C. Record the time from cutting the tip of the tail to stopping bleeding, the interval was called bleeding time (Kogushi et al. 2011). Heparin was used as positive control, and 0.9 % saline as model control.

### FXa Inhibition Assay

Chromogenic substrate method was used to measure the FXa-inhibiting activity. Inhibition was determined by FXa residual activity on the chromogenic substrate BIOPHEN CS-11 (S-2222) (22), which liberates the chromophoric group pNA (*p*-nitroaniline) (van Wijk and Smit 1984). Different concentrations of the peptide (5–60 mg/ml) were incubated with FXa for 30 min at 37 °C in PBS (pH 8.34). The reaction was initiated by adding the substrate into the peptide solution (Ibrahima et al. 2001). The absorbance at 405 nm was recorded every 0.5 min in 5 min. In A–t curve, the curve slope was considered as reaction rate (v).

 $$ \hbox{Inhibition rate} \, (\%) = {\frac{{ {{\text{V}}_{ 0}}
{{\text{ - V}}_{\text{i}} }  }}{{\text{V}}_{ 0} } }\,{\times}\,
100\,{\%}, $$ V_0_ is the rate of PBS (pH 8.34, model control), and V_i_ is the rate of peptide.

### Molecular Docking

To study the FXa inhibition mechanism of the peptide, Molecular Operating Environment (MOE, version 2009.10) was applied to predict how the peptide interacts with FXa (PDB ID: 2UWP). The good docking was determined using Molecular Mechanics Generalized Born Interaction Energy (MM/GBVI) in the MOE software. The MM/GBVI represents non-covalent interactions between the receptor and the ligand including the van der Waals energy, coulomb energy, and generalized Bonn implicit solvent interaction energy (Labute 2008).

### Statistical Analysis

Data are shown as mean ± SE for the number of experiments indicated, and ANOVA followed by Tukey’s tests were used for statistical comparison of the data. In all analyses, *P* < 0.1 (or *P* < 0.01) was considered as statistically significant.

## Results

### Purification of the Peptide

**Fig. 1:**
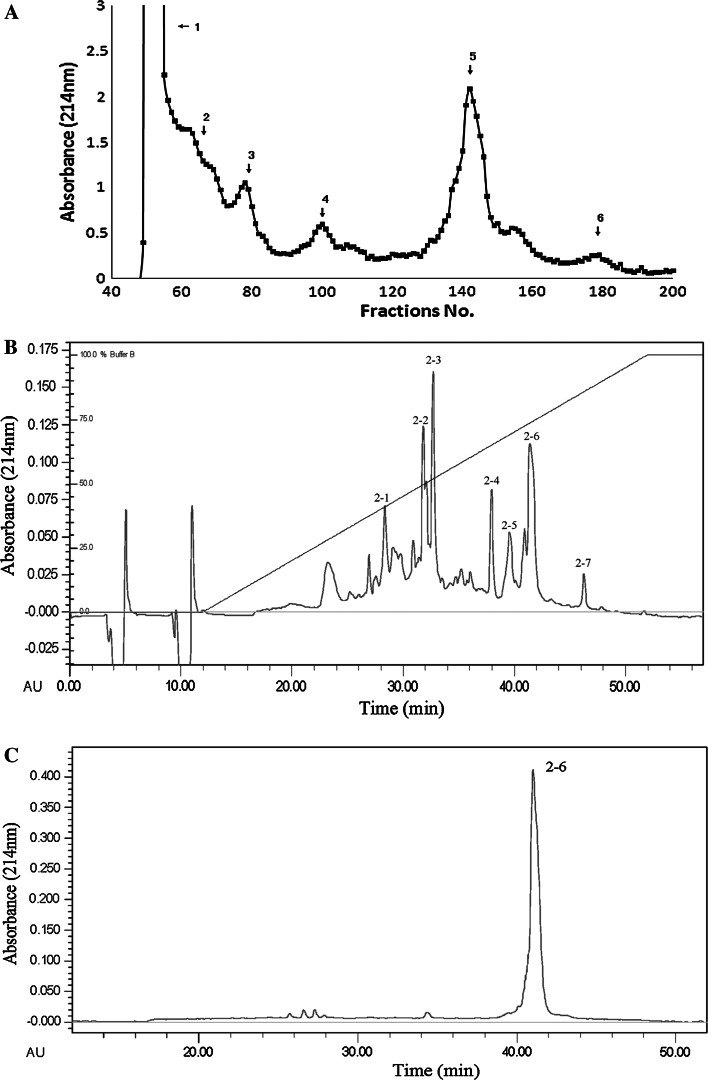
Purification of TNGYT from centipede venom. **a** *S. subspinipes mutilans* venom was separated by Sephadex G-50 column. **b** The faction 2 after Sephadex G-50 column was separated by RP-HPLC on a C18 column. **c** The fraction C-3 after RP-HPLC on a C18 column was separated by another C18 column. The final purified peptide was designated as TNGYT

The crude venom was separated into six fractions by Sephadex G50 column (Fig. 1a). Fractions 1, 2 and 3 showed aPTT and PT activity. Fraction 2 was then separated by RP-HPLC on a Lichrospher C18 column (Fig. 1b). Fraction 2–6 showed aPTT and PT activity. This active fraction was then purified by another C18 column (Fig. 1c) to give the final purified peptide.

### Determination of Molecular Mass and Peptide Sequence

**Fig. 2:**
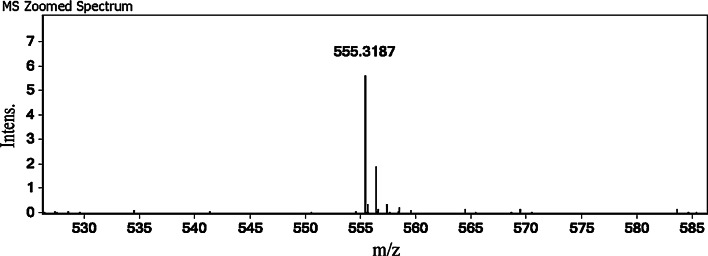
The molecular mass of TNGYT determined by ESI–MS. The molecular mass of purified TNGYT is 554.3 Da

The molecular mass of the purified peptide was determined to be 554.3 Da by ESI-MS (Fig. 2) and the primary structure was identified as Thr-Asn-Gly-Tyr-Thr (TNGYT) by Edman degradation. The molecular mass of TNGYT by ESI-MS matched the theoretical molecular mass (554.6 Da) deduced from amino acid sequence of TNGYT. By BLAST (http://blast.ncbi.nlm.nih.gov/) search, the peptide showed no similarity with any other known peptides or proteins in *S. subspinipes mutilans* (taxid: 251420). Although a few proteins from *Streptococcus parasanguinis* and *Leptosphaeria maculans* contained the fragment TNGYT, there was no single peptide fragment as TNGYT in the protein or peptide database.

**Fig. 3:**
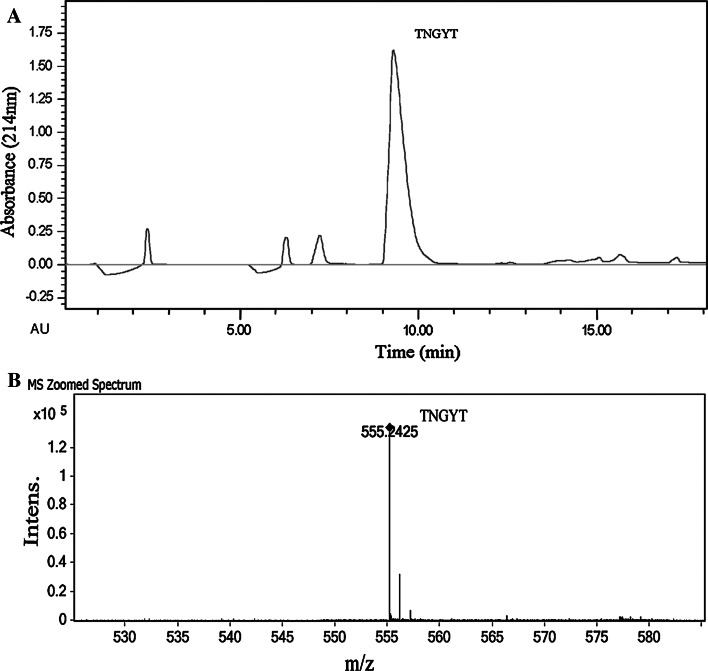
Purification of the synthetic TNGYT. The synthetic peptide solution was further purified by RP-HPLC on a preparative-scale C18 column (**a**). The purity of synthetic TNGYT was confirmed by ESI–MS (**b**)

### Peptides synthesis

All peptides used for the following bioactivity assays were synthesized by the Fmoc (*N*-[9-fluorenyl]-methoxycarbonyl) chemistry in solid-phase synthesis. The synthesized peptides were purified by RP-HPLC. The main peak was pooled (Fig. 3). The purity was higher than 95 % analyzed by HPLC. It was identical to the natural one confirmed by ESI-MS.

We utilized MS/MS to confirm the sequence of the synthetic peptide. As we expected, the result indicated that the synthetic peptide has the sequence of TNGYT (Fig. 1 in the Supplementary Data). Then, we compared the biological activity of the natural peptide and the synthesized peptide using aPTT and PT assays. The result indicated that the two peptides have the same biological activity (Table 1 in the Supplementary Data).

### Assay of aPTT and PT of TNGYT

For in vitro coagulation assays, TNGYT prolonged the aPTT and PT in a dose-dependent manner. It can prolong the aPTT clot time from 33.3 to 57 s and PT clot time from 12.7 to 23.3 s, respectively, at the concentration of 8 mg/ml (Table 1). The action of TNGYT was lower than the positive control heparin (1 mg/ml).

**Table 1:** APTT and PT assay in vitro

Concentration (mg/ml)	Sample	APTT (s)	PT (s)
0	0.9 % saline	33.3 ± 1.5	12.7 ± 1.2
1	Heparin	>60.0**	>60.0**
1	TNGYT	36.7 ± 1.5*	14.7 ± 0.6*
2	TNGYT	44.0 ± 2.0**	15.3 ± 1.5*
4	TNGYT	44.0 ± 1.0**	17.7 ± 0.6**
8	TNGYT	57.0 ± 2.0**	23.3 ± 1.5**

**P < 0.01, *P < 0.1, compared with 0.9 % saline

For ex vivo coagulation assays, when the mice were treated with 10 or 20 mg/kg TNGYT, no significant alterations were observed in the PT. However, increases in the aPTT were observed in both 5 and 24 h after drug administration (Table 2).

**Table 2:** APTT and PT assay ex vivo

Time (h)	Dose (mg/kg)	Sample	APTT (s)	PT (s)
5	0	0.9 % saline	49.0 ± 3.6	15.7 ± 1.5
	10	TNGYT	59.0 ± 2.0*	16.7 ± 0.6
	20	TNGYT	65.7 ± 3.1**	18.0 ± 1.0*
24	0	0.9 % saline	47.0 ± 1.0	17.7 ± 0.6
	10	TNGYT	49.3 ± 1.5*	18.7 ± 1.5
	20	TNGYT	51.3 ± 2.3*	18.3 ± 0.6

**P < 0.01, *P < 0.1, compared with 0.9 % saline

### Clotting Time In Vivo

In comparison with the control group, 20 and 10 mg/kg TNGYT group could significantly prolong whole blood clotting time (*P* < 0.01) as positive control heparin did. This indicated that TNGYT had anticoagulant effects. (Table 3).

**Table 3:** Effect of TNGYT and TGNYT on clotting time in mice

Code	Sample	CT (s)
Model control	0.9 % saline	273.2 ± 38.6
Positive control	Heparin (20 mg/kg)	718.8 ± 60.15**
TNGYT (10 mg/kg)	TNGYT (l0 mg/kg)	389.0 ± 35.74**
TNGYT (20 mg/kg)	TNGYT (20 mg/kg)	463.0 ± 48.8**

**P < 0.01, compared with model control (0.9 % saline)

### Bleeding Time In Vivo

Compared to the model control group, 20 and 10 mg/kg TNGYT groups could significantly prolong the bleeding time (*P* < 0.01). At equal dose (20 mg/kg), the bleeding time of heparin was longer than that of TNGYT (Table 4). It implied that the risk of bleeding by TNGYT treatment was lower than that of heparin.

**Table 4:** Effect of TNGYT and TGNYT on bleeding time in mice

Code	Sample	BT (s)
Model control	0.9 % saline	315.2 ± 33.4
Positive control	Heparin (20 mg/kg)	804.3 ± 87.7**
TNGYT (10 mg/kg)	TNGYT (10 mg/kg)	447.5 ± 78.1**
TNGYT (20 mg/kg)	TNGYT (20 mg/kg)	549.7 ± 60.9**

**P < 0.01, compared with model control (0.9 % saline)

### FXa Inhibition Assay

**Fig. 4:**
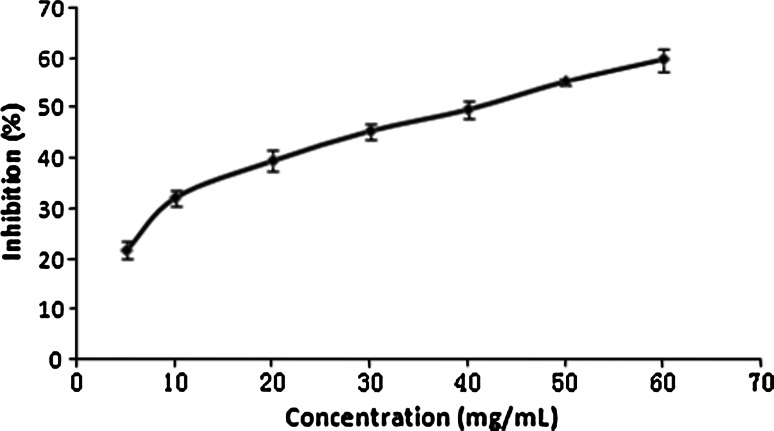
FXa inhibition curve of TNGYT. TNGYT inhibited the activity of FXa in a dose-dependent manner with an IC_50_ of 41.14 mg/ml

As shown in Fig. 4, TNGYT inhibited the activity of FXa in a dose-dependent manner with an IC_50_ of 41.14 mg/ml. TNGYT at a concentration of 60 mg/ml could inhibit FXa activity by 59.75 %.

### Molecular Docking

**Fig. 5:**
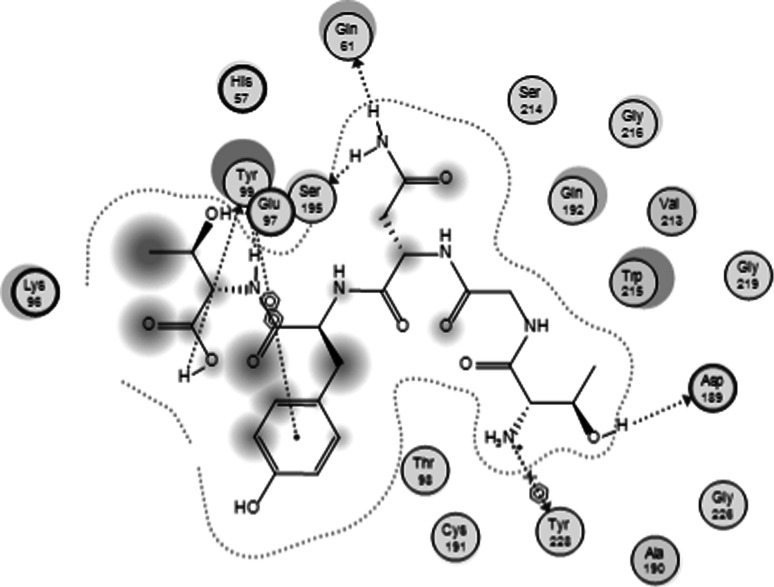
Molecular docking of TNGYT with FXa. TNGYT bound to Asp189, Ser195 and Tyr228 residues of S1 and Tyr99 residue of S4 of FXa

FXa contains a deep S1 pocket and a box-like S4 pocket at the active site. S1 pocket consists of Asp189, Ser195 and Tyr228 residues while S4 consists of Tyr99, Phe174 and Trp228 residues. Potential FXa inhibitors usually bind to both S1 and S4 pockets which are connected in L-shape (Rai et al. 2001). As shown in Fig. 5, it was found that TNGYT binds to Asp189, Ser195 and Tyr228 residues of S1 and Tyr99 residue of S4 at the MM/GBVI value of −17.696 kcal/mol. This docking result supported the result of FXa inhibition assay and confirmed that TNGYT is an FXa inhibitor.

## Discussion

The centipede contains many bioactive components, including 5-hydroxytryptamine, histamine, lipids, polysaccharides, and various enzymes (e.g. proteinases and esterases) (Knysak et al. 1998; Gomes et al. 1983). They have been widely used as traditional folk medicine to treat thrombotic diseases in China (Pemberton 1999) for several hundred years. You et al. (2004) isolated a 25 kDa serine protease from *S. subspinipes mutilans*, which demonstrated fibrinolytic activity by converting human Glu-plasminogen to activated plasmin. Wu et al. (2009) found centipede acidic protein (CAP) significantly suppress the development of atherosclerosis, improved the hemorrheological disturbances and histopathological changes in the atherogenic diet fed rat model. We previously isolated an anti-thrombotic peptide with primary structure of peptide is Ser-Gln-Leu (SQL) from *S. subspinipes mutilans* (Kong et al. 2013).

Centipede venoms contain a variety of different components with diverse functions (Mohamed et al. 1983). Ren et al. (2006) isolated an antibacterial peptide named scolopendrin I from the venom of *S. subspinipes mutilans*. González-Morales et al. (2009) isolated a phospholipase A_2_ from the venom of *S. viridis Say*. Peng et al. (2010) reported the structural and functional characterization of two antimicrobial peptides (scolopin 1 and -2) identified from the venoms of *S. subspinipes mutilans*. Rates et al. (2007) identified more than 60 proteins/peptides in *S. viridicornis nigra* and *S. angulata* venoms by a proteomic approach. Liu et al. (2012) further purified and characterized 40 proteins/peptides from crude venom of *S. subspinipes dehaani*. The purified proteins/peptides showed different pharmacological properties, including platelet aggregating, anticoagulant, phospholipase A_2_, trypsin inhibiting, voltage-gated potassium channel, voltage-gated sodium channel, and voltage-gated calcium channel activities. Yang et al. (2012) identified 26 neurotoxin-like peptides from the venom of *S. subspinipes mutilans*. In the present study, the pentapeptide TNGYT was isolated from *S. subspinipes mutilans* by a combination of gel filtration and reverse-phase HPLC.

Generally, the venoms from some snake, parasitic and blood-feeding animals, such as snake, tick, bat and leech are considered rich resource to discover new anticoagulant agents. Han et al. (2008) purified an anticoagulant serine protease named as magnvesin from the wasp venom of *Vespa magnifica*. Xu et al. (2000) isolated two anticoagulants from five-pace snake (*Agkistrodon acutus*) venom. Though centipede venom contained varied peptides and proteins, such as antimicrobial peptides (Peng et al. 2010), hemolytic peptide (Ren et al. 2007). To our best knowledge, it is the first peptide identified from *S. subspinipes mutilans* venom with anticoagulant and FXa-inhibiting activities.

The coagulation cascade is a dynamic and complicated process triggered by endothelial and/or plaque damage and consists of the extrinsic and intrinsic pathways. FXa is a trypsin-like serine protease that sits at the junction of both the extrinsic and the intrinsic pathways. FXa, in combination with its co-factor Va, converts prothrombin to thrombin, the final enzyme produced in the coagulation cascade. Thrombin activates platelets and also converts fibrinogen to fibrin, which leads to clot formation. Thrombin is also known to amplify its own production by activating factor XI to factor XIa which, in turn, leads to a further activation of FXa, thus leading to the further propagation of thrombin. Since FXa sits at the critical juncture of both pathways, its role as a regulator of thrombin generation is very critical in controlling the haemostatic network. So, the purified TNGYT could prolong the aPTT and PT both in vitro and ex vivo and whole blood clotting time possibly by the mechanism of inhibition of the FXa activity.

The active site of the FXa is mainly divided into four “pocket”: S1, S2, S3, and S4. S1 determines selective binding; S2 is small, and generally studied with S4; S3, located in the edge of S1, is completely exposed to the solvent environment; There are three ligand-binding domains in S4, named “hydrophobic cartridge (hydrophobic box)”, “cationic hole (cationic hole)” and the water point (water site). FXa inhibitor usually binds to Asp189, Ser195 and Tyr228 of S1 and Tyr99, Phe174 and Trp228 of S4 (Rai et al. 2001). TNGYT bound to Asp189, Ser195 and Tyr228 residues of S1 and Tyr99 residue of S4 mainly by side-chain interaction. Thus, the docking study revealed that TNGYT could inhibit the activity of FXa, but also suggested that the relatedly low potency of this natural peptide may result from its incomplete binding to FXa. Optimization of TNGYT into a drug molecule should aim to complement extra domains for full binding to FXa.

So far, the molecular mass of the known FXa inhibitors which belong to protein and peptide has been more than 5 kDa. *Ac*-AP-12 (Jiang et al. 2011) from the esophageal glands of adult *Ancylostoma caninum* was a 9.1 kDa anti-coagulation peptide with FXa inhibition activity. Amblyomin-X (Batista et al. 2010) was a single-chain protein with the molecular mass of 13,491 Da. Nymphal FXa inhibitor (Ibrahim et al. 2001) isolated from the camel tick *Hyalomma dromedarii* was a protein with the molecular mass of 15 kDa. FXa inhibitor named AduNAP4 (Gan et al. 2009) with 104 amino acids including a predicted 23-residue signal peptide, was from the human hookworm, *Ancylostoma duodenale*. The pentapeptide TNGYT in this study was the smallest peptide which could inhibit the activity of FXa. Although it was easier for preparation, the inhibition rate of TNGYT was lower than FXa inhibitors mentioned above. Further modification and transformation are needed to improve the FXa inhibition activity of TNGYT.

We could not rule out other underlying mechanisms that lead to TNGYT actions. Obviously, the peptide showed higher potency in animal studies compared to in vitro assays. At the same dose of 20 mg/kg, TNGYT produced about 50 % of the effects in prolonging the clotting time compared to heparin after i.v. injection to mice, while the potency of the peptide was at least 8-folded lower than heparin with in vitro assay. The superior result from animal studies is encouraging to further develop this natural peptide into drug entity. Nevertheless, it also warrants further mechanism studies to examine the different outcomes from in vitro and in vivo experiments. Animal study is more complicated setting than in vitro assays and it gives opportunities for the drug to interact with endogenous molecules as well as to be transformed chemically. It is possible that TNGYT gains extra potency when interacting with other molecules or being transformed into other molecules in vivo. We do not intend to resolve the mechanism in this first study of this novel peptide, but will focus on the mechanism study in the future.

In conclusion, this is the first report that an FXa inhibiting peptide was isolated from centipedes venom. The activity assay showed this novel peptide of TNGYT can anticoagulate in both in vitro and ex vivo assays and inhibited the activity of FXa in a dose-dependent manner. According to the results of molecular docking, the possible mechanism is that TNGYT binds to four amino acids residues within the active site of FXa.
